# Allegheny County Medical Society

**Published:** 1890-04

**Authors:** 


### ALLEGHENY COUNTY MEDICAL SOCIETY.

SPECIAL MEETING, FEBRUARY 19th, 1890.

W. S. FOSTER, M. D., PRESIDENT, IN THE CHAIR.

### TRACHEOTOMY FOR FOREIGN BODY.

DR. MURDOCH.—A little boy was brought to the West Pennsylvania Hospital recently, who five days before was lying on his back with a grain of corn in his mouth; he took a violent paroxysm of laughter, suddenly gave a very long inspiration and immediately was seized with a paroxysm of coughing and strangling. The parents, who knew that the boy had the corn in his mouth, surmised it had gone "the wrong way" and into his windpipe, and commenced, as is customary with people in such cases, to slap him on the back, and, not succeeding in relieving his paroxysm of coughing, they sought assistance from their family physician, who came and gave the child an emetic; the child vomited profusely, but was not relieved. He continued to cough, had violent paroxysms that night and all the following day, and then still further relief was sought, and every effort was made to dislodge the grain of corn from the windpipe. The child was inverted, shaken and slapped violently on the back; still other emetics were given but no relief came, and the father brought the little fellow to the hospital. He then presented every appearance of a child suffering from œdema of the lungs. The skin of the face was livid. The child was evidently in great distress, breathing with considerable difficulty, and every few moments he would be taken with violent paroxysms of coughing. On examination of his chest I was unable to detect any abnormality in either lung by auscultation or percussion. But from the history of the case, and from the other symptoms, I thought we were warranted in opening the windpipe. Before putting the child under an anæsthetic I inverted him and shook him, but the corn was not dislodged from its position. He was then anæsthetized with chloroform, and that procedure was repeated without good effect. An incision was then made into the trachea; the second, third and fourth rings of the trachea were divided, and when the forceps were introduced into the trachea, the child gave a violent inspiration and then a violent expiration and at once the corn came up from below and appeared at the opening. I made an effort to grasp it with the forceps but failed, and it went back into the trachea. Then by introducing a pair of narrow-bladed forceps to try to get the corn, the irritation of the forceps seemed to excite another paroxysm of coughing; the boy made another inspiration and after it an expiration, and the corn was thrown away across the room, lodging some eight or ten feet from the boy. It was picked up and found to be a very large grain. It must have been three-fourths of an inch in length by half an inch wide and a fourth of an inch thick, and it was in a swollen condition, and evidently had it remained much longer in the boy, it would have commenced to germinate. The case demonstrates the value of the operation of tracheotomy. I believe that if this body had been larger and more spherical, it would have probably lodged in the larynx. That is the usual way children choke. If it is a spherical body, or a body like a piece of meat, it will be impacted in the larynx, and unless the child is relieved at once he necessarily perishes. When the body is angular, as a grain of corn, if lodged in the larynx, sufficient air can pass to sustain life. In all cases we are not so successful as I was in this one. Frequently, when the windpipe is opened, the surgeon is unable to get the foreign body. It may be impacted in one of the bronchial tubes, and even if it does not escape at the time of the operation, the chances of its escaping from an opening in the trachea are much greater than they would be for it to escape through the larynx.

### DEAFNESS WITHOUT APPARENT LESION.

DR. ALLYN.—A man in a mill was assisting a man much larger than himself at a roll; a link of chain swung around and struck the larger man, breaking his nose and crushing his face, but simply pushing the smaller man to one side. This man showed no symptoms of prostration and presented all the appearances of being uninjured with the one exception that, as he was taken up, he was absolutely deaf to all noises. Going over all the points, I failed to elicit one point further than the fact that he could not hear. He had no aphasia, there was no paralysis of any of the muscles of the body, of the eyes or face. There was no paralysis of taste. The ear drums were intact, the membrane being perfectly translucent and of the proper color, and no hemorrhage known in the case at any time. It possesses the simple fact of there being absolute deafness in both ears, caused by merely being knocked over and falling on the side of the head. I do not know what progress the case will make; it is of recent origin.

### TREATMENT OF COMPOUND FRACTURES INVOLVING JOINTS.

DR. MCCANN.–I would like to talk about the treatment of compound fractures involving the joints, such as are attended by more or less destruction, not only of the bony tissues, but also the soft parts in the vicinity of the injury. Such injuries result commonly, as they have fallen under my observation, from two causes: first, accidents which happen to brakemen in coupling cars, in which the elbow is caught between the "drawheads" or "deadwoods" of a pair of cars. In putting in the link to make the coupling or in attempting to drop the pin, the elbow is caught directly between either the drawheads or the deadwoods. The result of this is an extensive fracture involving the elbow joint with extensive laceration and bruising of the soft tissues.

The second form of accident is that which involves the ankle joint, and in which the foot is caught either beneath the wheel or pinched by the brake-block. In one case, still under my care, the man fell between the trucks, his foot falling so that not the crown of the wheel but the flange passed across the outer and dorsal surface of the foot opening the ankle joint but not cutting through the tendo Achillis, though tearing the skin as far as the inner edge of that tendon. In another case, the accident involved the limb a little higher up, also opening the joint and crushing the astragalus. In the past, efforts to save such limbs usually resulted in a secondary amputation or in the death of the patient. The method of treatment is certainly a very important element in the history of these cases. Under the old *régimé*, the treatment usually adopted consisted in a sort of perfunctory cleansing of the wound, the application of carbolized oil or carbolic solution; the limb was placed in the position deemed most favorable in the eyes of the surgeon, and the reparative powers of nature were trusted to either cure the foot or to demonstrate the utter impossibility of saving it, if the patient did not die in the effort to find out whether or not his foot should be cut off. Within the past few years this has been modified, and the practice now (and I presume it is so all over the world) is to be guided by the extent of the injury. If the blood vessels and nerves are not involved, even if the bones be extensively crushed, an effort should be made to save the part, and this effort is comparatively simple, or rather the principles upon which it should be carried out are simple. First, cleanse the wound thoroughly, remove everything, fragments of bone, of devitalized skin, of wood or iron, everything foreign or liable to be septic. Then the limb should be thoroughly dressed antiseptically after being carefully washed in some solution, and the one I resort to is bichloride of mercury 1 to 1,000 or 1 to 2,000; the limb is then put up in an antiseptic dressing carefully but loosely applied, so as not to constrict but to protect the wound. If there is any tendency for the tissues to fall into such a shape that there will be pockets, I have no hesitancy in making counter openings, and introducing whatever number of drainage tubes may be necessary to secure proper discharge for the wound secretions. Now, having done this, the limb is placed on a splint, care being taken that there is no constriction of any part, that there is no tight bandage, no application which can in way interfere with the arterial circulation, or obstruct the return or venous circulation. The limb is elevated so as to favor the return circulation, and then dry heat is applied externally to all the dressings. The first dressing should be of sublimated or iodoformed gauze; borated or carbolated cotton is also applied to simply protect the wound by placing around it a sufficient amount of absorbent material, to exert a very moderate degree of elastic compression and to prevent constriction. Now, under this treatment you will either discover at the end of twenty-four hours that your limb is saved or is absolutely lost, and in the meantime you have protected your patient in the event of gangrene attacking the limb as a result of the traumatism. You have protected your patient against sepsis; and even if gangrene does occur, it does not spread with the rapidity it invariably assumes when the wound becomes septic. You have nothing to fear from the occurrence of that acute, spreading gangrene, the "gangrene of inflammatory sepsis," which has been the curse of surgery in the past. Usually at the end of twenty-four hours the first dressing should be changed, and it has been my habit to again cleanse the wound thoroughly to pass a stream of some antiseptic fluid, usually the 1-2000 bichloride solution, through the drainage tubes. Usually you will find one or two of them filled with coagulated blood. This should be removed, and if the opening is large enough it need not be replaced. The second should be applied just as the first. After this second dressing, it is usually unnecessary to replace the dressing for seventy-two hours or longer. A finger or toe should always be left uncovered, by which you can ascertain the condition of the extremities. If the toes or fingers continue warm when you expose them, and the capillary circulation perfect, you have nothing to fear. Now, under this treatment, if infection does not occur, the wound surfaces are not irritated by septic material. Suppuration does not occur. The discharges which flow from the wound are trifling in amount. The wound itself is comparatively painless. If there be dead portions, and usually there are, dead fragments of bone, dead shreds of skin or of bruised muscles remaining in the wound, of course they are foreign bodies; but the process of separation between the dead portion and the living goes on kindly and without suppuration. Without going further into the details of treatment, I may state that this plan should be carried on until the whole surface of the wound is cicatrized. The dressing does not require changing more than once in four or five days. Now to close this matter, I may state that of all the cases I have treated, five have involved the ankle joint, two of them were complicated by fractures of leg bones, one of them with the splitting of the tibia for eight inches. In all, the joint was widely laid open, extensive damage had been inflicted upon the bones with great laceration and contusion of the foot and of the tissues around the ankle. In another instance, one in which the foot was caught under the flange of the wheel by the patient falling between the trucks, the flange travelled up along the outer surface of the foot opening the ankle joint, crushing through the bones of the foot, so that when the stitches which had been injudiciously applied were cut, the crushed portion of the foot dropped apart.

Now under the method of treatment which I have advocated, thorough antiseptic cleansing, thorough draining, the use of loosely but thoroughly applied antiseptic dressings, this foot has been saved, and the man will soon be able to walk upon it.

DR. BUCHANAN.—I think the society is under an obligation to Dr.

McCann for the practical and excellent manner in which he has laid down the rules for the treatment of these injuries. I believe the general principles he has enunciated are the accepted ideas on the subject, and they are very well established to-day ; and for that reason I think we are disposed to take for granted that because Dr. McCann and certain other men, who see a very great amount of railroad and other surgery, do adopt these rules of conduct, their adoption is universal. I think this is a mistake, and I think we can very profitably stop and repeat these rules as Dr. McCann has done for us this evening. The most unexpected results, I think, can frequently be achieved by careful systematic and methodical repair of injuries. I exhibited to this society some time ago a case of complete excision of the ankle with excellent motion, in which there had been extensive laceration and crushing of the joints. Since that time I have attended another case in which the ankle, and indeed the whole posterior and middle part of the foot were so crushed that I hardly felt justified in asking the man to allow me to attempt to save it, but the man was urgent in his desire and would not permit the subject of amputation to be discussed; he knew the foot could be saved, and he gave me no option in the matter; and somewhat against my judgment I excised the ankle-joint, put the foot together, and the result is that to-day the man walks very well with a cane, and will soon dispense with it. The astragalus, a portion of the os calcis, the lower extremities of the leg bones and some pieces of the other tarsal bones were lost. The lower end of the tibia was wired to the lower part of the os calcis, and the soft parts of the foot were stitched together and dressed, as Dr. McCann has so well described. The patient recovered without a bad symptom. The wire was removed some weeks afterward. I have also treated a case lately with Dr. Huselton and my father, in which there was a laceration of the tissues about the ankle joint, fractures of both malleoli, and complete disarticulation and projection of the leg bones, which plowed into the earth. This patient had his joint cleansed and articulated; the inner malleolus was fixed into place with a buried wire, and there was no reaction whatever; afterward the man made an uninterrupted recovery.

TRAUMATIC SECTION OF BOTH TENDONES ACHILLIS.

I have also recently treated a case of injury to one ankle joint, and to the leg on the opposite side, by a mowing machine. The man who subsequently became the patient stepped in front of the cutting bar of a mowing machine and struck the horses; the animals responded immediately; the cutting bar of the machine cut off the tendo Achillis, passed directly through the ankle, cutting off both malleoli, both posterior arteries, and the tendon of the posterior tibial muscle. On the other side the section was higher up; it passed through the tendon of Achilles and cut a piece off the tibia. Both the tendones Achillis were sewed with cat-gut, four stitches; the other stitches were put in place, and the usual antiseptic dressings applied without drainage. The man's wounds healed without any reaction or discharge whatever, and he walks well to-day without support. The union of the tendons was perfect. I think it is well to emphasize the fact that so long as there is circulation in a limb, an effort should be made to save it, and I think also that such an effort will usually be successful. In one particular, only, I would make some difference in the treatment from that laid down by Dr. McCann. I think that as time goes on I see less use for drainage tubes. When I first commenced to employ this treatment, I used a drain wherever I had a chance. There might have been some fluid, and there probably was some fluid blood running during the first twenty-four hours, but afterward it was coagulated; after the primary dressing of the wound, I never washed it out,and in three or four days removed the tube with the clot in it, and have never seen any reason to change that practice; but I can believe that drainage tubes are more used than is necessary, and that if we succeed with our antisepsis and apply proper pressure, we need not be afraid of any accumulation of fluid in a wound. I have also seen that if, in a soft cavity there has been a serous accumulation because proper pressure has not been applied, such accumulation gives rise to no serious inconvenience, and when evacuated the walls of the cavity, collapse and unite without further discharge. I would therefore think that, instead of endeavoring to put in all the drainage tubes possible, I would limit them as a matter of convenience. The tube does no harm if properly used, but I think it is as usually applied unnecessary.

DR. HUSELTON.—In the main I agree withDr. McCann. I want to say that the amount of damage done to the skin in the case of a crush wound is no criterion of the amount of damage done to the interior parts, bones, muscles, nerves and vessels, and in accordance with my experience I think that where a hand, a wrist joint, an arm or an elbow joint, is caught between the bumpers of a car, the couplers or the deadwoods, sufficiently to produce a fracture, even if not compound, the best thing to do is to take that limb off; if you do not you will regret it and have to do it afterward. I have had considerable experience with injured ankle joints in railroad accidents. One of them was produced by the flange of a wheel; the joint was open so that I could put my fingers in. This I dressed antiseptically, cleansing it of all foreign material, washed it out, put it up antiseptically with a splint, without a drainage tube. I do not use drainage tubes very often, and I think I shall use them less often hereafter. This man got well and left my care before he had perfect locomotion in the joint; it had entirely healed, there was considerable motion; he was making a pretty good stagger at walking. In another case in which the right limb was crushed from the knee to the ankle joint, the left ankle was open so you could see into it also. In this case I amputated the right leg. I treated the ankle joint as in the previous case described, and the man to-day has a perfect ankle joint and a good wooden leg, and is walking around as well as any of us. I do not change the dressing so frequently as does Dr. McCann; sometimes do not remove the dressing for a week. I am governed entirely by the condition of the part and by the patient's condition as to temperature and pulse. If there are symptoms indicating the necessity of removing the dressing, I remove it, otherwise I leave it alone.

DR. RIGG.—I would like to say a word in reference to the class of wounds spoken of by the gentlemen who have preceded me. In 1882, I treated my first compound fracture of the elbow.

It was caused by an accident to a young man at work in a coal mine; he fell in front of a wagon, his foot caught in a frog, throwing the wagon off the track, the wagon running over his arm and elbow joint about the middle third. The superficial veins were lacerated, the arteries intact. The bleeding was controlled with little trouble. Since it was my first case of the kind I took two physicians with me when I went to see the case a second time. They insisted on amputation. I removed at that time, I think, a half-dozen small pieces of bone; the young man insisted on leaving the arm as it was, believing there was a fairly good arm left. My plan was to treat it antiseptically, strictly so, and I placed it on an incline, put no tight dressings to it at all, and had my dressings laid in such a way that I could at my own pleasure remove a portion of them to see what was going on beneath. I might say that the shortening, as nearly as I could make it out, was three and a half inches. I continued on this plan of treatment, and I may say without drainage tubes, until the arm was well and there was fair motion. There was never perfect motion. The humerus was very much thickened by reason of the fragments of bone not being adjusted closely. As to drainage tubes, I have used few. The last two cases of amputation I had, no drainage tubes were employed; they were amputations of the breast, and I believe them to be fairly good cases to test the value of a drainage tube, there being much surface made bare; and in both cases there was primary union throughout, something I have never seen where a drainage tube was used, and I feel that if the antiseptic treatment is followed out strictly, a drainage tube in the majority of cases is of little or no value, and oftentimes a little disadvantage.

DR. MCCANN.—I want to say a word in regard to two or three of the criticisms. In the first place, I do not think that because a limb, elbow or wrist has been caught between a deadwood or drawhead, it necessarily requires amputation. I can show any one who is curious enough to come to the West Penn Hospital instances where not only the bones were crushed, but the soft parts also, that were saved.

As to drainage tubes, I do not like to introduce a foreign substance into a wound, would rather get along without it if I could; but for a crush involving the tissues of the joint or the muscles there must necessarily be a large amount of fluid secretions.

Now I do not care whether they are pent up underneath the skin without any access to air, they are likely to generate poisonous and irritating leucomaines, which cause local irritation and constitutional infection. We all know that before the days of antisepsis the tight closure of wounds was far less favorable in its results than the open method. Who can be sure when he closes his wound, that he has rendered it aseptic? And if he has accomplished this, there may be floating in his patient's blood germs capable of infecting the wound.

DR. BUCHANAN.—There are two points on which I wish to speak. The first is the possibility of saving a limb that has been fairly and squarely caught between the bumpers, with fracture of the bones. I will cite a single instance. About six or eight months ago, I was called with my father to see a little girl whose arm had been caught between the bumpers of two freight cars; the arm was crushed, wholly crushed from the wrist to the elbow. The vessels were not injured, but there were multiple fractures of both bones, and considerable pieces of bone had to be taken out. I think it safe to say more than half the muscular tissue was crushed off. The skin was entensively lacerated. The arm was shapeless. I do not think any injury could deserve the name of crush better than this one. It was so evidently a serious injury that her parents and friends and all persons who saw it thought the arm must be amputated. This arm was not amputated; it was thoroughly cleansed and put together, dressed with a straight splint; on no other kind of splint could the arm be kept in the semblance of an arm. It united without any suppuration, as is usual in such cases when antiseptically dressed, and while the child has not a beautiful arm, nor a very straight arm, she has a hand and a wrist and an elbow-joint that are almost as useful as before. Now, as to drainage tubes, I think it is hardly fair to bring up the surgery of ancient times to prove that drainage tubes are good things. We will all admit that the essential object of a primary drain is to eliminate fluids which might become the breeding place for micro-organisms. And I am further satisfied that if we can exclude local infection of the wound, we need have no fear of its infection through the general circulation.

DR. GREEN.—As I understand Dr. McCann, in regard to drainage tubes, in applying an antiseptic dressing it is true of course that nobody can be positive that he leaves the wound without any danger of sepsis; I care not how carefully a wound may be washed, it is simply impossible to remove all the putrescible matter; a portion of it is bound to remain in the wound. It seems, therefore, the part of wisdom to provide means for its discharge.

